# Speech-evoked cortical activities and speech recognition in adult cochlear implant listeners: a review of functional near-infrared spectroscopy studies

**DOI:** 10.1007/s00221-024-06921-9

**Published:** 2024-09-21

**Authors:** Reed Farrar, Samin Ashjaei, Meisam K. Arjmandi

**Affiliations:** 1 Department of Psychology, University of South Carolina, 1512 Pendleton Street, Columbia, SC 29208 USA; 2 Department of Communication Sciences and Disorders, University of South Carolina, 1705 College Street, Columbia, SC 29208 USA; 3 Institute for Mind and Brain, University of South Carolina, Barnwell Street, Columbia, SC 29208 USA

**Keywords:** Cochlear implants, Speech-evoked cortical processing, fNIRS, Individual differences

## Abstract

Cochlear implants (CIs) are the most successful neural prostheses, enabling individuals with severe to profound hearing loss to access sounds and understand speech. While CI has demonstrated success, speech perception outcomes vary largely among CI listeners, with significantly reduced performance in noise. This review paper summarizes prior findings on speech-evoked cortical activities in adult CI listeners using functional near-infrared spectroscopy (fNIRS) to understand (a) speech-evoked cortical processing in CI listeners compared to normal-hearing (NH) individuals, (b) the relationship between these activities and behavioral speech recognition scores, (c) the extent to which current fNIRS-measured speech-evoked cortical activities in CI listeners account for their differences in speech perception, and (d) challenges in using fNIRS for CI research. Compared to NH listeners, CI listeners had diminished speech-evoked activation in the middle temporal gyrus (MTG) and in the superior temporal gyrus (STG), except one study reporting an opposite pattern for STG. NH listeners exhibited higher inferior frontal gyrus (IFG) activity when listening to CI-simulated speech compared to natural speech. Among CI listeners, higher speech recognition scores correlated with lower speech-evoked activation in the STG, higher activation in the left IFG and left fusiform gyrus, with mixed findings in the MTG. fNIRS shows promise for enhancing our understanding of cortical processing of speech in CI listeners, though findings are mixed. Challenges include test-retest reliability, managing noise, replicating natural conditions, optimizing montage design, and standardizing methods to establish a strong predictive relationship between fNIRS-based cortical activities and speech perception in CI listeners.

## Introduction

Over 1.5 billion individuals experience hearing loss worldwide, constituting nearly 20% of the global population (WHO, 2023). Nearly 48 million of these individuals have severe to profound hearing loss (WHO, 2023), placing them at greater risk for spoken communication difficulties (Arjmandi and Behroozmand 2024; Dalton et al. 2003; Lin et al. 2011; Strawbridge et al. 2000), anxiety (Littlejohn et al. 2022; Strawbridge et al. 2000), poor cognitive abilities (Uchida et al. 2019; Wayne and Johnsrude 2015), and social isolation (Mick et al. 2014; Shukla et al. 2020; Strawbridge et al. 2000; Weinstein and Ventry 1982). Cochlear implants (CIs) are the most successful neural prostheses that provide partial access to sounds in the environment for individuals with severe to profound hearing loss, with a global reach of one million recipients (Zeng 2022). CIs bypass the damaged or non-functional hair cells within the inner ear and directly stimulate the surviving spiral ganglion neurons of the auditory nerve using electrical pulses. The CI sound processor generates these pulses by analyzing the speech signal, dividing it into specific frequency channels, adjusting pulse amplitude via each electrode’s signal envelope, and mapping channel data to the CI electrodes. On average, CIs improve speech recognition score in quiet to approximately 62% (Holden et al. 2013), although individual outcomes following implantation exhibit high variability (Arjmandi et al. 2022a; Caswell-Midwinter et al. 2022; Holden et al. 2013), with many CI listeners performing poorly, specifically in background noise (e.g.Arjmandi et al. 2022a).

Despite extensive research, the sources of this large individual variability and the extent of their contributions are not yet completely understood, posing challenges in the development of targeted interventions for speech outcomes improvement. Studies have predominantly focused on examining the impact of peripheral processing factors, such as the suboptimal quality of the interaction between individual CI electrodes and their target auditory neurons (i.e., electrode-neuron interface (ENI) quality) and the limited number of electrodes compared to thousands of hair cells in a healthy cochlea, on speech recognition in CI listeners (e.g., Arenberg, 2010; Arjmandi et al. 2022a; Cucis et al. 2021; DiNino et al. 2016; Donaldson et al. 2015; Hughes and Stille 2008; Arjmandi et al. 2021). The goal has mainly been to understand how these factors may affect a CI listener’s ability to integrate spectral information in speech, which relates to the frequency/spectral components of speech sounds (e.g., fundamental frequency, formant frequencies, spectral envelope), and temporal information in speech, which refers to the timing aspects of speech sounds (e.g., phoneme duration, voice onset time, temporal envelop), for speech recognition in quiet and in background noise. CI listeners have limited access to spectral information in speech, although temporal envelope modulations are transmitted by CI with reasonable accuracy (Chatterjee and Peng 2008; Nie et al. 2006; Steinmetzger and Rosen 2018). While these aspects of cochlear implantation do contribute to outcome variability, a large portion of around 30–40% remains unexplained (Arjmandi et al. 2022a; Caswell-Midwinter et al. 2022; Holden et al. 2013).

Alternatively, evidence from several studies suggests that cortical processing can contribute to variability in speech perception outcomes (Defenderfer et al. 2021; Lawrence et al. 2018; Levin et al. 2022; Steinmetzger et al. 2022; Scheperle and Abbas 2015a,b). Individual adult CI listeners may experience differential adaptation and reorganization in their brains in response to auditory information through CIs, which may result in distinct functional changes in processing speech (Fullerton et al. 2023; Ratnanather et al., 2020). Various neuroimaging techniques exist for measuring differential cortical processing evoked by listening to speech, but a promising technique is functional near-infrared spectroscopy (fNIRS). fNIRS is a functional neuroimaging technique that measures cortical changes in hemoglobin concentration by analyzing differential near-infrared light absorption. It uses a series of source-detector pairs to transmit and receive light at specific wavelengths, thereby measuring the degree of light absorption. Variations in this light absorption indicate cortical activity in response to tasks such as speech perception. The present review summarizes prior studies to identify current knowledge and gaps about speech-evoked cortical activities in adult CI listeners as measured by fNIRS. It compares these findings between actual CI listeners and NH listeners, as well as with NH individuals when listening to CI-simulated speech versus natural speech. For CI-simulated speech, studies used a vocoder simulation in NH listeners (NH-VOC) to compare the results with those obtained when listening to natural, unprocessed speech (NH-NS). It also reviews the findings on the association between these cortical activities and behavioral speech perception outcomes.

A greater understanding of how individual differences in speech-evoked cortical activation are associated with variations in speech recognition scores is essential for the development of fNIRS-based objective measures of speech perception in CI listeners. These measures can eventually assist in targeted CI optimization and the development of rehabilitation approaches focused on enhancing the neural representation of spectral and temporal cues of speech at the cortical level. They can also allow us to study how factors such as the variation in the quality of ENI (e.g., Arenberg, 2010; Arjmandi et al. 2022a; Donaldson et al. 2015; Hughes and Stille 2008) and the degree of CI channel interaction (Cucis et al. 2021; Throckmorton and Collins 2002), which occurs when neural excitation patterns of adjacent CI channels excessively overlap, distort the cortical representation of spectral information in speech. Since the primary objective of cochlear implantation is achieving open-set auditory-only speech recognition in everyday listening scenarios, this review focuses on the fNIRS findings on the cortical processing of speech in adult CI listeners based on auditory-only speech stimuli without the assistance of other sensory information such as visual cues.

The speech-evoked cortical activities can be measured using various neuroimaging techniques, such as electroencephalography (EEG), magnetoencephalography (MEG), or functional magnetic resonance imaging (fMRI). However, most face challenges that prevent fully effective use for studies of speech perception in CI listeners. The top priority of a neuroimaging technique is the safety of the participant (Alberalar et al. 2023). Another important aspect of an effective neuroimaging technique is ease of use for the participant. Neuroimaging methods such as fMRI fail to meet these criteria in this particular clinical population. CIs have internal magnets and ferromagnetic components, including a coil for transmitting data from an external processor to the implanted components. During fMRI, these ferromagnetic implants, exposed to electromagnetic fields or radiofrequency energy, may experience heating, induce currents, or become displaced. The magnet and coil also interact with the electromagnetic fields in MRI scanners, causing interference that can disrupt data transfer and create large ferromagnetic artifacts that distort MRI signal, especially in regions nearest to the device (Schröder et al. 2018). Malfunctions may occur due to demagnetization of the CI’s internal magnet by the imaging magnet (Alberalar et al. 2023).

Using EEG for CIs research also faces a similar problem in the form of an electrical artifact that greatly reduces usefulness of signal (e.g., Intartaglia et al. 2022). While analysis techniques exist to exclude these artifacts, there is not yet a consensus on the best practices for doing so (Intartaglia et al. 2022). Several works have used EEG to study speech perception in adults CI listeners at the cortical level (e.g., Zheng et al., 2023; Soshi et al. 2014; Han and Dimitrijevic 2020; Turgeon et al. 2014). While these studies show that EEG can provide valuable insights into cortical activity in response to speech, its poor spatial resolution limits the ability to pinpoint specific cortical regions of interest (ROIs) (e.g., Nunez et al. 1994). This limits EEG’s ability to.

identify how individual differences in the magnitude of speech-evoked cortical in certain ROIs relate to speech perception outcomes, highlighting the need for a technique with better spatial resolution such as fNIRS. fNIRS also offers a faster and more streamlined process compared to EEG. Although the focus of the present review is on fNIRS studies on speech-evoked cortical activities in CI listeners, future work could explore the potential benefit of integrating EEG with fNIRS on studying speech-evoked cortical activities in CI listeners. Positron emission tomography (PET) has also been used successfully without CI-related contraindications (Jansje et al., 2021), but it involves the use of invasive radioactive tracers, which are invasive, especially for monitoring purposes.

A promising alternative to these techniques is fNIRS (Bauernfeind et al. 2016), which has several advantages both specific and non-specific to CI listeners. fNIRS is a completely safe and non-invasive technique for CI listeners that does not suffer from electrical or ferromagnetic artifacts. It is also portable, relatively cost-effective, generates minimal noise during recording, and offers moderate spatial resolution in the 1–2 cm range (e.g., Bortfeld 2019). While the spatial resolution of fNIRS is lower than that of fMRI, it is considerably better than EEG (e.g., Wilcox and Biondi 2015). It also provides moderate temporal resolution, making it better than fMRI but poorer than EEG in this aspect (Wilcox and Biondi 2015). These qualities make fNIRS a practical choice for research involving speech perception in CI listeners (e.g., Bauernfeind et al. 2016; Saliba et al. 2016).

**Fig. 1:**
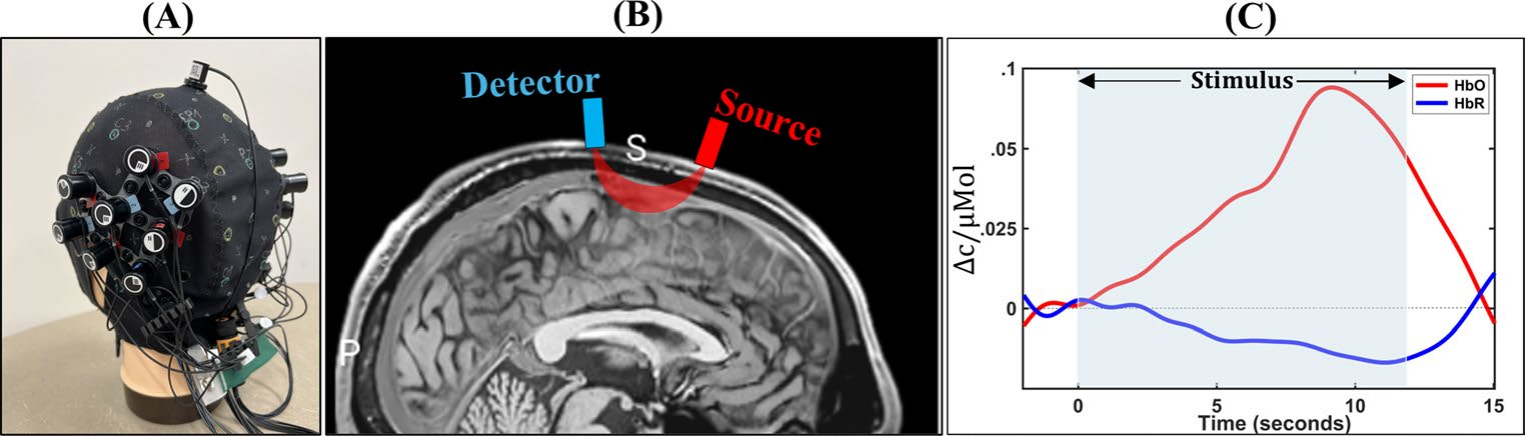
Panel (**A**) shows an fNIRS setup fitted on a model head, primarily covering the auditory cortex. The red tags indicate the light sources and blue tags indicate the detectors. Panel B shows a sagittal view of the brain with a visualization of a source (in red), detector (in blue), and the banana-shaped channel that passes through the cortex between them (in semi-transparent red). Panel C shows an example of an average hemodynamic response evoked by a speech stimulus in one channel across multiple trials. The estimates of cerebral oxygenated (HbO in red line) and deoxygenated hemoglobin (HbR in blue line) are shown as the change in concentration of hemoglobin in micromoles (*ΔC*/*µmol*)

Here, we provide a brief overview of the principles of fNIRS neuroimaging. fNIRS operates on the principle that energy metabolism increases in specific brain regions during tasks such as listening to speech. This process creates an increased oxygen demand, which leads to raised oxygenated hemoglobin (HbO) concentration and lowered deoxygenated hemoglobin (HbR) concentration in the regions during activity (e.g., Bauernfeind et al. 2016). fNIRS neuroimaging takes advantage of this process by measuring the extent to which near-infrared light is absorbed by hemoglobin. fNIRS measurements are taken by a set of optodes held to the head by a cap that keeps them in contact with the scalp (see Panel A in Fig. 1). The optodes are divided into two types, namely sources and detectors. Sources in the fNIRS apparatus send targeted wavelengths of light into the brain that are absorbed primarily by either HbO or HbR (e.g., Abtahi et al. 2016). Then, detectors measure the intensity of light that returns to their locations on the scalp. The paths that light travels between sources and detectors are termed channels, and they are the result of a portion of light scattering through the tissues of the head to the location of the detector through a path called the “*photon banana*” (see Panel B in Fig. 1). The depth of a channel can be increased by lengthening the space between optodes, but limitations in spatial resolution prevent fNIRS from accurately measuring deeper than approximately 5–8 mm below the cortical surface (Karim et al. 2012; Stoppelman et al. 2013).

Light absorption is determined by first measuring the raw, or unprocessed, light intensity that returns to the detector. The Modified Beer-Lambert Law is used to convert the measured intensity into estimates of changes in cerebral HbO and HbR concentrations based on light attenuation (Izzetoglu et al. 2005). Following removal of motion artifacts, systemic noise, and other components of the signal that prevent direct observation of evoked neural activity (Dans et al. 2021), the average task-evoked hemodynamic response for a channel or ROI is calculated and visualized (see Panel C in Fig. 1 for an example hemodynamic response). While there are many options available for processing and the optimal method can vary based on study design (Dans et al. 2021), an important component for removing extracerebral noise is the use of short channels. These involve placing a detector near enough to a source that captures data from the scalp without measuring concentration changes in the cortex. This information is subtracted from the signal obtained from long channels to provide a better reflection of cortical activity.

fNIRS has been successfully used by some studies to capture group-level differences in speech perception outcomes between CI and NH listeners and between NH-VOC and NH-NS conditions (e.g., Levin et al. 2022; Zhou et al. 2018), but many of the findings from these studies regarding speech-evoked activation are mixed (Bortfeld 2019; Defenderfer et al. 2021; Levin et al. 2022; Steinmetzger et al. 2022; Zhou et al. 2018). Furthermore, only a limited number of studies have investigated the association between individual speech-evoked cortical differences and behavioral speech perception scores. Advancements in precision and reliability in capturing individual differences in speech-evoked cortical activities might eventually help locate focal points in cortical regions for refining speech perception based on developing effective CI signal processing techniques (e.g., Soleymani et al. 2018), CI programming strategies (Caswell-Midwinter and Arenberg 2023), and auditory rehabilitation (Dornhoffer et al. 2024), especially in complex listening environments.

Measurements of adult CI listeners’ cortical activities in response to speech are better understood in the context of results obtained from those with NH. It is widely agreed that the superior temporal gyrus (STG) is heavily involved in understanding speech (Hamilton et al. 2021; Lin et al. 2019; Norman-Haignere et al. 2015; Obleser et al. 2010; Oganian et al. 2023; Yi et al. 2019), and its activation is associated with spectro-temporal analysis of speech (Hickok et al. 2011; Oganian et al. 2023), as well as phonetic qualities of words and sentences (Creutzfeldt et al. 1989). The middle and inferior temporal gyri are also associated with comprehension of spoken language (Creutzfeldt et al. 1989). The left inferior frontal gyrus (LIFG) has demonstrated a significant role in both phonological and semantic processing of language (Liakakis et al. 2011; Rogers and Davis 2017). These findings demonstrate that cortical activity in the auditory cortex and associated areas is related to various aspects of speech perception, providing a basis for analyzing the association between listening to speech and the corresponding neural activity in CI listeners.

Prior studies with other neuroimaging modalities have found some differences between the cortical activation of CI and NH listeners in several auditory-associated regions in the brain. These studies have observed greater STG activation in response to speech among CI listeners compared to NH listeners (Naito et al. 2000; Sherafati et al. 2022), and this effect has been extended to surrounding regions such as the middle temporal gyrus (MTG) (Naito et al. 2000; Sherafati et al. 2022), inferior frontal gyrus (IFG) (Naito et al. 2000; Sherafati et al. 2022), and anterior cingulate gyrus (ACgG) (Naito et al. 2000). These findings provide a knowledge base against which fNIRS findings can be compared, and they give context to experiments that have examined how the relationship between speech-evoked cortical activation and speech perception ability differ between CI and NH listeners. In adult CI listeners, greater magnitude of neural activation in the primary auditory cortex (e.g., Green et al. 2005) and the auditory association area (e.g., Fujiki et al. 1999) has been linked to higher speech perception scores using PET. A similar link was observed using EEG for NH listeners, in whom higher N1 amplitudes were associated with better speech perception in noise (e.g., Parbery-Clark et al. 2011). Together these findings suggest that greater speech-evoked activation in the STG corresponds to greater speech perception ability, although the findings may vary depending on the specific tasks, their cognitive demand, and regions of interest. Despite prior findings, the field still lacks sufficient studies based on fNIRS neuroimaging in determining specific cortical ROIs and their contributions to speech-evoked cortical activities and speech perception outcomes. This gap hinders a comprehensive understanding of the relationship between speech perception ability and speech-evoked activity in adult CI listeners.

This review is primarily motivated by gaps in our understanding of fNIRS-based speech-evoked activities in adult CI listeners, how these activities differ between adult CI listeners and NH listeners, and how they may explain speech recognition performance among CI listeners. We specifically analyzed and synthesized prior findings for evidence on (a) fNIRS-based speech-evoked cortical activities in adult CI listeners, (b) group level differences in cortical activity in response to processing speech between CI and NH listeners, (c) how these speech-evoked cortical activities are associated with speech recognition performance, and (d) the extent to which individual differences in cortical activity in response to auditory speech stimuli within the cortical auditory system is associated with individual differences in speech perception scores in adult CI listeners. To our knowledge, this is the first review that synthesizes findings from fNIRS studies in adults CI listeners for these purposes. We also highlight and discuss gaps in knowledge and methodological challenges associated with effectively utilizing fNIRS to measure speech-evoked cortical activities in adult CI listeners and to capture individual differences in the cortical processing of speech.

## Method

A comprehensive review was conducted to identify relevant studies that have explored speech-evoked cortical activity in adult CI listeners using fNIRS and examined the relationship between this activity and speech perception outcomes. A list of key words related to our research questions was compiled. Prominent among these were “adult cochlear implant users/listeners/recipients”, “functional near-infrared spectroscopy or fNIRS”, “cortical or speech-evoked cortical activity”, and “speech perception or speech recognition.” We used these keywords to search for and identify relevant articles from the PubMed, EMBASE, Scopus, Web of Science, ProQuest, and Google Scholar databases. No publication date exclusions were made except that studies published after the search date were not eligible for consideration. A total of 68 unique papers were identified with this process. Next, screening occurred based on the content of papers. We sought to include all papers that used fNIRS to measure speech-evoked cortical activity in adult actual CI listeners and/or in NH individuals listening in NH-VOC conditions versus listening in NH-NS conditions. Exclusion criteria were developed to screen and remove papers. The criteria and the number of excluded papers is as follows: no adult CI listeners tested or no NH-VOC condition used (23), did not use fNIRS to measure cortical activity (6), cortical activity not evoked solely by auditory speech (9), secondary sources such as review papers or editorials that did not align with the goals of this review (17), and publications that were only abstracts (3). The screening process led to the exclusion of 58 papers, leaving 10 major studies for review, which are summarized in Table 1.

**Table 1:** Summary of fNIRS studies on speech-evoked cortical activities in adults CI listeners. Information includes experiment design, type of speech stimuli, listening conditions (quiet or noise), participants information, fNIRS setup, regions of interest, behavioral measure of speech perception, and key findings. CI: cochlear implant; fNIRS: functional near-infrared spectroscopy; NH: normal hearing; NH-VOC: normal hearing’s response to vocoded speech; HINT: hearing in noise test; BKB: bench-Kowal-Bamford; CNC: consonant-Nucleus-Consonant; CUNY: City University of New York; OLSA: Oldenburg sentences test; SRT: Speech Recognition threshold; STG: Superior temporal gyrus; DLPFC: Dorsolateral Prefrontal Cortex; MTG: middle temporal gyrus; IFG: Inferior Frontal Gyrus; MFG: Middle Frontal Gyrus; STS: Superior temporal sulcus; PrG: Precentral Gyrus; PoG: Postcentral Gyrus; IPL: Inferior Parietal lobe; HbO: oxygenated hemoglobin response, HbR: deoxygenated hemoglobin response

Study	fNIRS Experiment, Stimuli, and Listening Condition	Design	Participant Information	fNIRS Setup Information	Regions of Interest	Behavioral Measure of Speech Perception	Major Findings
Fullerton et al. 2023	Blocks of 4 concatenated IEEE sentences in quiet	Block design	*N* = 31 (17 NH, 14 CI); NH: age at testing 53–79 years (mean 70 years); CI: Age at testing 54–80 years (mean 68 years); postlingually deafened adults; 4–68 years (mean 19.8 years) bilateral deafness; >= 12 months CI experience	52 long channels; 17 sources, 16 detectors; 30 mm long channel separation; no short channels stated	Left and right STG	---	• The left and right STG showed significant speech-evoked activation. • No CI-vs.-NH group comparison was made.
Levin et al. 2022	Unprocessed and noise-vocoded sentences in three conditions: speech in quiet, speech in speech-shaped noise, and noise-only	Block design	*N* = 44 (15 NH, 15 NH-VOC, 14 CI); age at testing 18–44 years; CI: prelingually deafened adults; age at identification of hearing loss between 1 month and 5.5 years old; 7 early implanted ( < = 3 years old) and 7 late implanted ( > = 4.5 years old); age at testing 18–40 years	33 long channels, 8 short channels; 16 sources, 16 detectors; 35 mm long channel separation; 12 mm short channel separation	Right DLPFC, left DLPFC, pars triangularis Broca’s, bilateral STG, bilateral MTG, left fusiform gyrus, right fusiform gyrus	Sentence recognition thresholds in speech-shaped noise	• Significant cortical activities were found in the right DLPFC and bilateral MTG for speech in quiet, and in the bilateral MTG and left fusiform gyrus for speech in speech-shaped noise. • CI listeners showed smaller cortical responses bilaterally in the MTG than NH listeners for speech in quiet and in noise, which was mainly driven by data from poor-performers. • For all NH, NH-VOC, and CI listeners combined, mean beta values of GLM analysis for HbO data evoked by speech in noise were negatively correlated with sentence recognition thresholds in noise in the left (*r* = -0.356)^*^ and right MTG (*r* = -0.495)^*^.
Shader et al. 2022	Segments from a children’s story in quiet	Block design	*N* = 12 (12 CI); age at testing 38–78 years (mean 62.3 years); CI: Postlingually deafened adults; Age at onset of hearing loss 8–62 years; CI experience between < 1 and 34 days	44 long channels, 8 short channels; 16 sources, 16 detectors, 8 short-channel detectors; 30 mm long channel separation, 8 mm short channel separation	Left IFG left Heschl’s gyrus, right Heschl’s gyrus, bilateral planum temporale, bilateral superior occipital gyrus, bilateral cuneus, bilateral middle occipital gyrus	---	• fNIRS activity across all participants was only significant in the right Heschl’s gyrus. • Immediately after activation, CI recipients experienced auditory speech-evoked activation lateralized to the hemisphere contralateral to the side of implantation. For bilateral CI listeners, activation was present bilaterally.
Steinmetzger et al. 2022	Blocks of 20 concatenated vowels with a manipulable pitch contour	Block design	*N* = 20 (20 unilateral CI with NH in contralateral ear; single-sided deaf CI users); age at testing 26–78 years; CI: postlingually deafened adults; duration of deafness mean 10 years; duration of CI use mean 4 years and 3 months	36 long channels, 4 short channels;16 sources, 16 detectors; 30 mm long channel separation, 15 mm short channel separation	Bilateral STG	Freiburg monosyllabic speech intelligibility test in quiet	• For the CI ears, fNIRS activity reached significance level for the STG, left primary auditory area and the superior temporal sulcus. • fNIRS activity was larger along the right STG and near left primary auditory cortex for the NH ears compared to CI ears. • A negative correlation was observed between change in HbO concentration in the STG for the CI ears and monosyllabic word intelligibility scores (*r* = -0.48). Pitch contours evoke auditory cortex activity bilaterally, although this effect was stronger for NH ears than for CI ears.
Defenderfer et al. 2021	HINT sentences in quiet, vocoded, low-level speech-shaped noise, high-level speech-shaped noise, and high-level noise vocoded	Event-related design	*N* = 38 (38 NH-VOC); age at testing 18–30 years (mean 24.76)	13 long channels, one short channel; 4 sources, 8 detectors; 30 mm long channel separation, 10 mm short channel separation	Left MFG, left IFG, left MTG	Percent correct repetition of HINT sentences	• Cortical activity in the left MTG was stronger in response to speech in quiet compared to vocoded speech in quiet. This activity increased for vocoded speech in noise relative to vocoded speech in quiet. • Correlations between change in HbO concentration and HINT sentence repetition performance in the vocoded condition vs. quiet were negative in the left MTG (*r* = -0.398) and positive in the left IFG (*r* = 0.430).
Zhou et al. 2018	Blocks of 5 spondee words in quiet	Block design	*N* = 29 (15 CI, 14 NH); NH: age at testing 33–70 years (mean 53.5 years); CI: age at testing 46–79 years (mean 64.2 years); postlingually deafened adults; >12 months CI experience	46 long channels; 16 sources, 16 detectors; 30 mm long channel separation; no short channels stated	Bilateral STG Bilateral STS	CNC words in quiet; CUNY sentences in quiet and multitalker babble noise	• Cortical activities were observed on bilateral STG and bilateral STS in response to speech in quiet. • CI listeners displayed greater auditory speech-evoked activity in the left STG and the anterior temporal lobe bilaterally than NH listeners. • Speech recognition scores among CI listeners were negatively correlated with activity in the left middle superior temporal lobe and STG (*r* = -0.65) and the right anterior temporal lobe (*r* = -0.62).
Lawrence et al. 2018	Noise-vocoded Bamford-Kowal-Bench (BKB) sentences with varying intelligibility at levels of 0, 25, 50, 75 and 100% keywords correct	Event-related design	*N* = 23 (23 NH); age at testing 18–38 years (median 24.6 years)	44 long channels; 16 sources, 14 detectors; 30 mm long channel separation; no short channels stated	Bilateral STG, right PrG, right PoG, Bilateral IFG, right IPL, left IPL	Percent of keywords from vocoded BKB sentences repeated correctly.	• Significant activation was observed in the bilateral STG, right PrG, right PoG, bilateral IFG, and right IPL in response to vocoded speech. • fNIRS activation decreased monotonically in left and right superior temporal regions as intelligibility of the vocoded speech decreased. • In the LIFG, positive activation was observed in all conditions relative to silence. • fNIRS response amplitude contrast between 50% intelligibility and 0% intelligibility in the right STG was positively and significantly correlated with individual behavioral sentence recognition scores (*r* = 0.52).
Olds et al. 2016	Audiobook narration that was either normal speech, vocoded speech (channelized or scrambled), or replaced with environmental sounds	Block design	*N* = 67 (32 CI, 35 NH); NH: age at testing 24–65 years; CI: age at testing 23–86 years; postlingually deafened adults; 1 day − 12 years CI experience	31 long channels; 16 sources, 24 detectors; 30 mm to 33 mm long channel separation; no short channels stated	Bilateral STG	SRT, CNC word scores in silence, AzBio sentence recognition in quiet	• High-performing CI listeners showed greater bilateral STG activation from unprocessed speech than degraded speech, but poor-performing CI listeners had similar STG activation across all tested degradation levels.
van de Rijt et al., 2016	Reading aloud of children’s stories	Block design	*N* = 38 (33 NH, 5 CI); NH: age at testing 18–62 years (median 29 years); CI: age at testing 55–59 years (median 57 years); postlingually deafened adults; 5–19 years CI experience	2 long channels, 2 short channels; 4 sources, 2 detectors; 35 mm long channel separation, 25 mm short channel separation	Bilateral temporal cortices	---	• fNIRS can reliably capture temporal cortex responses to auditory stimuli.
Pollonini et al. 2014	Reading of a story under conditions of normal speech, noise-vocoded speech (both channelized and scrambled), and environmental noise	Block design	*N* = 19 (19 NH); age at testing 19–63 years	41 long channels, 29 short channels; 16 sources, 24 detectors; 15 to 21 mm short channel separation, 30 to 45 mm long channel separation	Bilateral primary auditory cortices	---	• Greater speech degradation was associated with lower auditory cortical activation with smaller brain network. • Normal speech reliably evoked the strongest responses within the auditory cortex. • fNIRS was more responsive to concentration changes in the right hemisphere than the left, which could have been caused by more shallow activity in the right auditory cortex.

*Note that higher sentence recognition threshold in Levin et al. 2022 corresponds to lower speech recognition scores, indicating poorer speech perception. Therefore, the negative *r* values reported for this study indicate a positive relationship between speech recognition scores and fNIRS activities

Some studies used simulated CI speech that involved processing speech stimuli with a noise vocoder (Pollonini et al. 2014; Olds et al. 2016; Lawrence et al. 2018; Defenderfer et al. 2021; Levin et al. 2022). Vocoded speech is produced by digital processing of a natural speech signal to emulate the auditory experience of CI listeners. This involves dividing the speech signal into several frequency bands/spectral channels (often 4, 8, 12, 16, 22, or 32 bands) using bandpass filters, extracting the amplitude envelope of each band through rectification and low-pass filtering, and using these amplitude envelopes to modulate a carrier signal such as white noise (noise- vocoded) or sinusoids (sine-vocoded) which has undergone through the same process (e.g., Arjmandi et al. 2021; DiNino et al. 2016). The modulated signals from each band are then combined to generate the final vocoded speech signal. This process reduces the spectral and temporal resolution of the speech to mimic the auditory limitations experienced by CI listeners. In experiments, vocoded speech stimuli are prepared using signal processing software, and NH participants are instructed to listen to degraded speech to simulate CI hearing (e.g., Defenderfer et al. 2021; Lawrence et al. 2018; Arjmandi et al. 2021). We use NH-VOC and NH-NS to refer to the findings from testing NH listeners when they listen to speech processed by a vocoder to simulate CI speech processing (NH-VOC), and when they listen to natural, unprocessed speech (NH-NS), respectively.

Table 1 presents these studies and their relevant information. From the final list of papers, 8 papers used block design experiments and 2 papers used event-related designs. The average number of long channels across studies was 29, ranging from 2 to 52, while short channels averaged 5, ranging from none to 29. Four studies did not use short channels. The potential advantages of using short channels are discussed in section “F. Major Challenges”. Studies used a variety of speech tasks. Eight studies presented sentences either as stories or discrete sentences (Defenderfer et al. 2021; Fullerton et al. 2023; Lawrence et al. 2018; Levin et al. 2022; Olds et al. 2016; Pollonini et al. 2014; Shader et al. 2022; van de Rijt et al., 2016), one study while listening to spondee words (Zhou et al. 2018), and another when listening to vowels (Steinmetzger et al. 2022). Three studies assessed speech recognition in noise (Defenderfer et al. 2021; Levin et al. 2022; Zhou et al. 2018). Four studies measured fNIRS activities in NH-VOC condition (Defenderfer et al. 2021; Lawrence et al. 2018; Levin et al. 2022; Pollonini et al. 2014). In terms of ROIs, the studies mainly focused on brain regions involved in auditory and speech perception, including STG, superior temporal sulcus (STS), MTG, middle frontal gyrus (MFG), fusiform gyrus, the dorsolateral prefrontal cortex (DLPFC), Heschl’s gyrus, occipital gyrus, planum temporale, IFG, inferior parietal lobe (IPL), precentral gyrus (PrG), postcentral gyrus (PoG), and overall temporal cortices. We discuss the findings in the following sections. Note that the findings from Heschl’s gyrus and fusiform gyrus should be interpreted with caution, as fNIRS face limitations in capturing data from these deep brain regions (e.g., Karim et al. 2012; Stoppelman et al. 2013).

### fNIRS-measured speech-evoked cortical activities in adult CI listeners

**Fig. 2:**
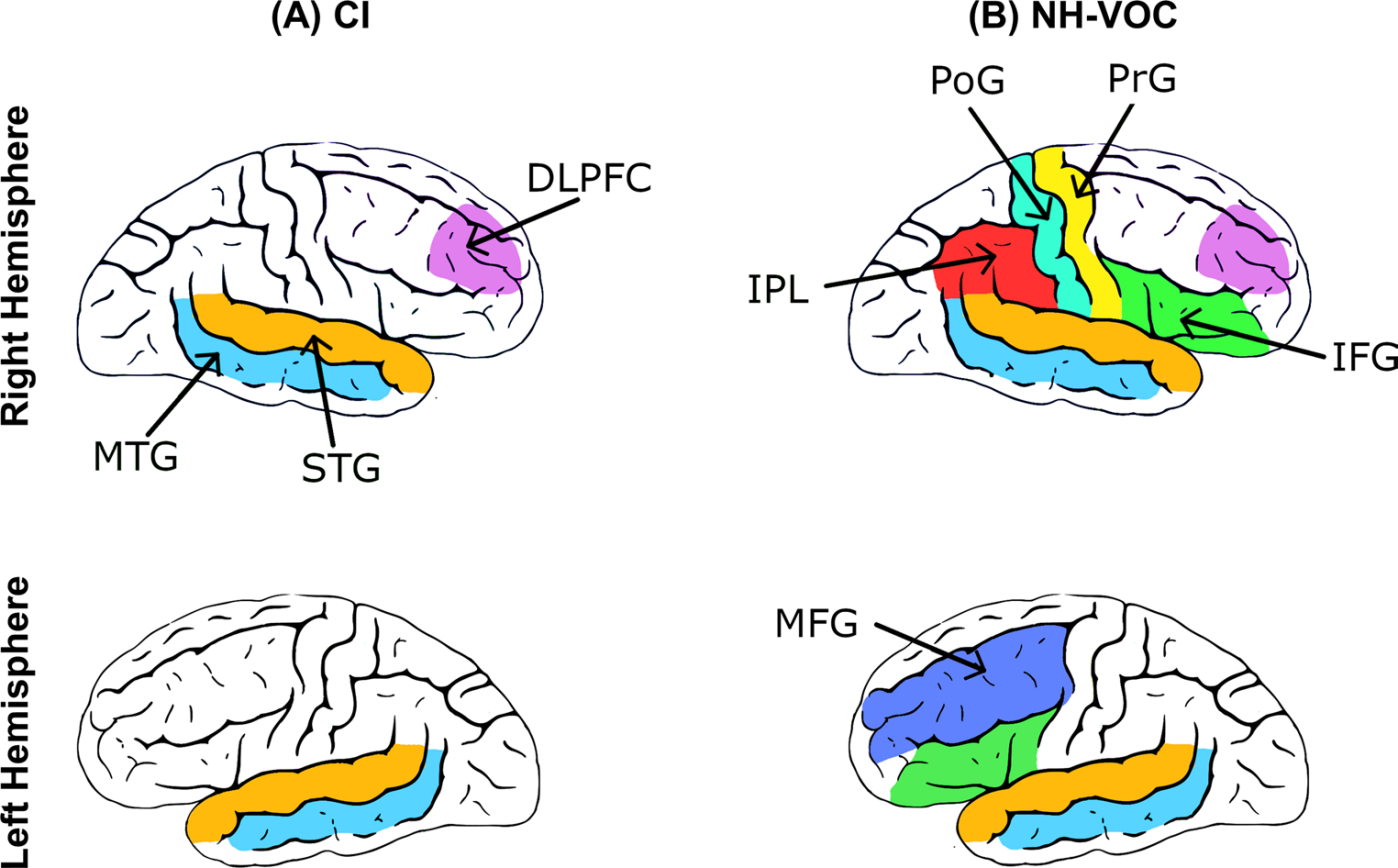
The approximate anatomical locations showed involvement in fNIRS-measured speech-evoked activities from adult CI listeners (panel **A**) and in NH-VOC conditions (panel **B**). MTG (in light blue): middle temporal gyrus; STG (in orange): superior temporal gyrus; DLPFC (in purple): dorsolateral prefrontal cortex; IPL (in red): inferior parietal lobe; PrG (in yellow): precentral gyrus; PoG (in cyan): postcentral gyrus; MFG (in dark blue): middle frontal gyrus; IFG (in green): inferior frontal gyrus. While the right Heschl’s gyrus and left fusiform gyrus also showed speech-evoked activity, they are not included in this figure because they are located deeper in the brain, which is potentially beyond the detection capabilities of fNIRS

We summarize findings from studies investigating speech-evoked cortical activities using fNIRS in adult CI listeners. These findings were based on testing actual CI listeners, comparing them to NH listeners, and evaluating results under NH-VOC or in comparison with NH-NS condition. Note that NH-NS refers to natural, unprocessed speech, which is equivalent to NH in this context. This distinction is made to facilitate comparisons between NH-VOC and NH-NS findings. The goal is to identify the current evidence on the anatomical distribution and magnitude of speech-evoked cortical responses in adult CI listeners. Figure 2 summarizes consistent patterns of speech-evoked cortical activity observed in CI listeners across studies. Note that the colors in Fig. 2 are only used to denote locations of ROIs in the cortex. They do not imply any differences in the magnitude of cortical activity in these regions, and these areas may not be active to the exact same extent. Any effects that were lateralized to a particular hemisphere were reported regardless of side of implantation or stimulation, unless otherwise stated. For adult CI listeners, the STG (Fullerton et al. 2023; Olds et al. 2016; Steinmetzger et al. 2022; van de Rijt et al., 2016; Zhou et al. 2018) and MTG (Levin et al. 2022) showed significant speech-evoked cortical activities bilaterally (panel A in Fig. 2). Heschl’s gyrus (Shader et al. 2022) and the dorsolateral prefrontal cortex (DLPFC in panel A in Fig. 2; Levin et al. 2022) in the right hemisphere and the left fusiform gyrus (Levin et al. 2022) showed significant activity in response to speech, although the findings in Heschl’s gyrus typically exhibit low specificity due to the low signal-to-noise ratio (SNR) in fNIRS signals in this deep brain region (Luke et al. 2021; Stoppelman et al. 2013).

Some studies included NH-VOC conditions to approximate cortical activity in response to listening to speech through CIs (Defenderfer et al. 2021; Lawrence et al. 2018; Levin et al. 2022; Olds et al. 2016; Pollonini et al. 2014). The findings from these studies are shown in panel B of Fig. 2. These studies showed significant bilateral fNIRS-measured cortical activities in the STG (Lawrence et al. 2018; Pollonini et al. 2014; Olds et al. 2016), MTG (Defenderfer et al. 2021), and IFG (Lawrence et al. 2018). In the left hemisphere, these responses were found to be significant in the middle frontal gyrus (MFG; Defenderfer et al. 2021), while, in the right hemisphere, the inferior parietal lobe (IPL), precentral gyrus (PrG), postcentral gyrus (PoG) (Lawrence et al. 2018), and DLPFC (Levin et al. 2022) showed significant cortical activities in NH-VOC conditions. Among these brain regions, the STG has been most frequently identified as activated in response to speech in actual CI listeners and in NH-VOC conditions. This might be because the STG has been more frequently studied as compared to other areas. While significant speech-evoked activation was found in the STG and MTG for both actual CI listeners and in the NH-VOC condition, a broader network of cortical involvement was reported in the NH-VOC condition. (panel B cf. panel A in Fig. 2). This broader cortical involvement in processing vocoded speech may be due to the additional cognitive and perceptual processing required to interpret vocoded speech. In fact, NH listeners are not accustomed to the degraded auditory signal presented by vocoded speech. As a result, they possibly engage more brain regions to process and understand the degraded speech signal. In contrast, CI listeners have often adapted to the specific auditory input provided by their implants over time. CI listeners in most studies in Table 1 had at least six months of experience hearing through their CI. This adaptation phase might explain the narrower network involved in processing speech in CI listeners compared to the NH-VOC condition.

Certain ROIs have shown varying degrees of involvement in speech perception, potentially influenced by the constraints of fNIRS. One study that examined speech-evoked activation in Heschl’s gyrus bilaterally found significant speech-evoked activation compared to baseline levels in only the right Heschl’s gyrus (Shader et al. 2022). Although no significant activities were found in the left Heschl’s gyrus, prior findings from other neuroimaging modalities observed significant involvement of this area in processing speech in individuals with hearing loss (Neuschwander et al. 2019; Ratnanather 2020; Smith et al. 2011). This discrepancy may be due to fNIRS’ limited sensitivity to this region, since Heschl’s gyrus is located deep in the lateral fissure (Luke et al. 2021). Another effect found by Shader et al. (2022) was greater activation in the Heschl’s gyrus contralateral to the side of implantation immediately following CI activation. Rather than indicating a preference for one hemisphere over the other, greater activity in the temporal region contralateral to the CI highlights the importance of taking both hemispheres into account when evaluating speech-evoked cortical activities and the lateralization of cortical speech processing (Boemio et al. 2005; Newcombe and Ratcliff 1973). Speech recognition outcomes can undergo significant changes for adult CI listeners as the duration of CI use increases following implantation, especially within the initial six months following implantation (Caswell-Midwinter et al. 2022; Holden et al. 2013). Further research is required to identify whether this effect persists or how it changes as the duration of hearing through a CI increases.

More nuance was also reported in the IFG, which exhibited more prominent speech-evoked activation in the left hemisphere despite showing a bilateral pattern in NH-VOC condition (Lawrence et al. 2018). Moreover, the finding of speech-evoked activity in the IFG is inconsistent, as certain studies have reported no such activity in this region in NH-VOC conditions and CI listeners (Defenderfer et al. 2021; Shader et al. 2022). This lack of speech-evoked cortical activation in the IFG reported by these studies is consistent with a finding from a NH-NS condition (Shader et al. 2021), which reveals that the IFG is not expected to show speech-evoked activation when measured by fNIRS. Potential sources of these mixed findings in the IFG could also be the variability among studies in their experimental designs and their numbers of short channels employed (see Table 1), as well as fNIRS’ limitations in capturing neural activities deep in the cortex. In addition, as outlined in Table 1, the available studies vary in characteristics of the study population, although differences in participants’ age at time of testing and comparison between actual CI versus a NH-VOC condition did not explain their differences in speech-evoked IFG activity (Defenderfer et al. 2021; Lawrence et al. 2018; Shader et al. 2022). Of these possibilities, variation in the use of short channels is likely the most influential due to their critical impact on the quality of fNIRS data (van de Rijt et al., 2016).

### Group-level comparison of speech-evoked cortical activities between CI and NH listeners and NH-VOC vs. NH-NS conditions

**Fig. 3:**
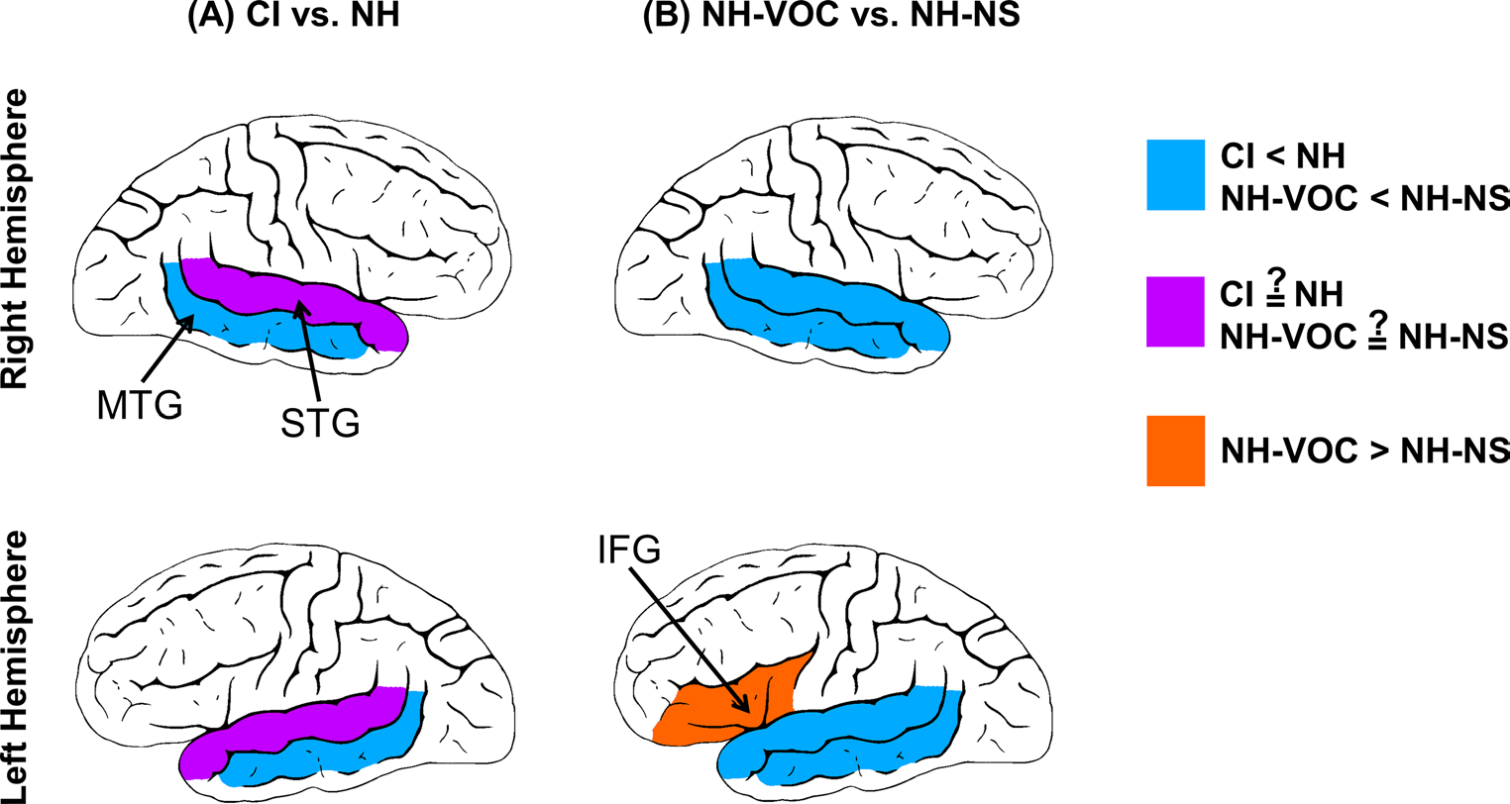
Patterns of speech-evoked activity based on comparisons made between CI and NH listeners (panel **A**) and between NH-VOC and NH-NS conditions (panel **B**). Cortical regions where at least one study found a significant difference in the magnitude of speech-evoked cortical activation between groups are illustrated. Note that all ROIs in this figure are labeled in Fig. 2. Some regions shown in Fig. 2 are not included here because no studies have examined or reported comparisons between CI listeners or NH-VOC condition and NH listeners for these regions. Blue color indicates weaker fNIRS activities in CI listeners compared to NH listeners or in NH-VOC conditions with varying degrees of signal degradation or compared to a NH-NS condition. Orange indicates the reverse pattern. Purple indicates mixed findings. “?” sign indicates mixed findings. STG: superior temporal gyrus; MTG: middle temporal gyrus; IFG: inferior frontal gyrus

Accurate and reliable identification of differences between CI listeners and NH listeners in anatomical location and magnitude of neural activities can help identify specific deficiencies or compensatory mechanisms in the neural processing of speech in CI listeners in response to speech. Comparing speech-evoked cortical activation patterns between CI and NH listeners can also be used to understand what neural mechanisms are unique to hearing speech through CIs. Six studies have reported a difference in neural responses to speech among actual CI listeners compared to NH individuals or in NH-VOC condition compared to NH-NS condition in certain brain regions (Defenderfer et al. 2021; Lawrence et al. 2018; Levin et al. 2022; Pollonini et al. 2014; Steinmetzger et al. 2022; Zhou et al. 2018). Panel A of Fig. 3 summarizes findings for actual CI listeners versus NH listeners, and Panel B summarizes results for the NH-VOC condition versus NH-NS condition. Further details of the relevant studies are presented in Table 1. Note that in Fig. 3, the colors indicate whether fNIRS activities were weaker (in blue), stronger (in orange), or mixed (in purple) in actual CI listeners (panel A) or NH-VOC condition (panel B) compared to NH-NS condition.

Activation in the STG has been studied the most, and three out of four studies found that NH listeners exhibit greater speech-evoked activation compared to actual CI listeners (Steinmetzger et al. 2022) and compared to NH-VOC conditions (Lawrence et al. 2018; Pollonini et al. 2014) in this brain region. These findings suggest that CI listeners may exhibit altered or diminished neural responses in the STG relative to NH individuals. The STG is an important brain region involved in processing auditory information and decoding the spectral and temporal information in speech (e.g., Hickok et al. 2011; Oganian et al. 2023). One major function in speech perception is listeners’ spectral resolution ability, which refers to their ability to perceptually distinguish between sounds with different spectral (frequency) components. Limited spectral resolution processing in CI listeners, resulting mainly from a small number of spectral channels and suboptimal ENI, may hinder the accurate processing and representation of various frequency information in speech signals (e.g., pitch, formant frequencies, and spectral centroids; Hazrati and Loizou 2012; Kasturi et al. 2002; Kokkinakis and Loizou 2011; Li and Loizou 2009; Oxenham and Kreft 2014; Parikh and Loizou 2005; Qin and Oxenham 2005). This could lead to suboptimal or deviated neural responses in the STG, potentially driving weaker speech-evoked activation when processing speech. Establishing this connection remains subject to ongoing investigation and requires further exploration.

Despite this common finding, one study found the opposite effect, reporting that actual CI listeners showed greater activation than NH listeners in the left STG (Zhou et al. 2018). This opposing finding might be the result of the use of a different type of stimuli, as Zhou et al. (2018) used CNC word stimuli in contrast to the use of story-reading (Pollonini et al. 2014), sentence (Lawrence et al. 2018), and vowel (Steinmetzger et al. 2022) stimuli in the studies that found greater speech-evoked STG activity among NH listeners compared to CI listeners. Overall, these findings suggest that the STG in adult CI listeners responds more weakly to speech compared to that of NH listeners.

More homogenous findings have been reported regarding neural activity in the MTG and the LIFG. In the MTG, speech-evoked cortical activation was stronger in NH individuals bilaterally compared to CI individuals (Levin et al. 2022) and compared to a NH-VOC condition (Defenderfer et al. 2021; see panels A and B in Fig. 3). As with the STG, decreased speech-evoked activities in the MTG, especially among CI listeners with poor speech recognition scores (Levin et al. 2022), may be attributed to limited spectral resolution ability of CI listeners (Arjmandi et al. 2022a; DeVries and Arenberg 2018; DiNino et al. 2016; Levin et al. 2022; Loizou and Poroy 2001). The opposite effect was observed for activation in the LIFG, where greater activation was found in NH-VOC condition as speech stimuli became more degraded (i.e., less natural) (Lawrence et al. 2018). See panel B in Fig. 3. The observed stronger activation in the LIFG in response to more degraded speech may indicate increased demand for a broader cognitive resource such as working memory when the received speech signal is degraded (e.g., Rogalsky and Hickok 2011). This expanded brain network could also be due to an attempt to compensate for the spectrotemporally-degraded sensory information by increasing activity beyond the auditory cortex or due to increased listening efforts in response to processing a degraded speech signal (e.g., Winn & Teece, 2021; Larsby et al., 2005; Zekveld et al., 2010). Together, these results suggest differences in cortical processing of speech between NH listeners and CI listeners, as well as between NH-VOC and NH-NS conditions in the STG, MTG, and LIFG. These findings suggest that CI and NH listeners may differ in bottom-up (sensory) and top-down cortical processing of speech, potentially due to differences in sound coding fidelity (i.e., degradation of incoming speech signal) and the impact of higher-level processes such as auditory working memory and executive function.

Overall, the results from these studies suggest that adult CI listeners exhibit weaker cortical activities in the MTG and STG compared to NH individuals, with the exception of one study that found the opposite trend in the STG (Zhou et al. 2018). Further support for this conclusion is provided by studies that simulate CI processing using vocoded speech (Levin et al. 2022; Defenderfer et al. 2021). Although these studies used vocoded speech to simulate various aspects of CI speech processing, these simulations do not replicate the long-term cortical changes that occur in actual CI listeners. Based on these simulated-CI studies, cortical activities diminish in both the MTG and STG and expand in the LIFG in a NH-VOC condition in contrast to a NH-NS condition.

### Relationship between speech perception and fNIRS-based speech-evoked cortical activities

Some fNIRS studies have used correlational approaches to examine the relationship between fNIRS-based cortical activities and behavioral measures of speech perception. A set of five studies (see Table 1) used correlational approaches to examine the relationship between fNIRS-based speech-evoked cortical activity and behavioral speech perception outcomes in actual CI listeners (Zhou et al. 2018; Levin et al. 2022; Steinmetzger et al. 2022) and in NH-VOC condition (Lawrence et al. 2018; Defenderfer et al. 2021; Levin et al. 2022). In the STG, two studies found a negative correlation between fNIRS activity and speech recognition scores in actual CI listeners in response to vowel (Steinmetzger et al. 2022) and spondee stimuli (Zhou et al. 2018), although this relationship was limited to the left hemisphere in response to spondees (Zhou et al. 2018). Their results indicated that decreased activity in the STG was associated with higher speech-perception scores. In contrast, Lawrence et al. (2018) found that better sentence recognition scores in NH-VOC listeners correlated with greater differences in fNIRS-measured right STG activity between listening to vocoded sentences with 50% intelligibility and vocoded sentences with 0% intelligibility. This positive relationship is consistent with evidence from Green et al. (2005), a PET study that showed greater activity in CI listeners with better speech perception ability. However, these findings from Lawrence et al. (2018) contradict the negative relationship observed by Steinmetzger et al. (2022) and Zhou et al. (2018). Steinmetzger et al. (2022) and Zhou et al. (2018) measured changes in HbO concentration in actual CI listeners using block design experiments, whereas Lawrence et al. (2018) based their results on changes in fNIRS amplitude in NH-VOC condition where listeners exposed to varying intelligibility levels in an event-related design. These differences in measurement reporting and experimental design prevent a direct comparison of these results. Overall, the scarcity of relevant fNIRS studies, particularly those using story stimuli in actual CI listeners, makes it difficult to identify the source of this disparity in findings.

In the MTG, a negative correlation between speech-evoked activity in the MTG and speech perception scores was found by Defenderfer et al. (2021) only in the left MTG. In contrast, Levin et al. (2022) found a positive correlation in the MTG bilaterally. The negative relationship in the MTG reported by Defenderfer et al. (2021) was in a NH-VOC condition in quiet, and the positive relationship in Levin et al. (2022) obtained across all three groups of NH, NH-VOC, and CI listeners in noise. This difference in listening conditions lends evidence to the idea that listening to speech in quiet may engage different cortical processes than listening to speech in noise, although it is also possible that the inclusion of NH and CI listeners into the relationship in addition to NH-VOC condition by Levin et al. (2022) played a role in finding a different correlation direction. In addition, findings from NH-VOC conditions have provided evidence of the involvement of brain regions beyond the auditory cortex. Defenderfer et al. (2021) found that better speech recognition scores were positively correlated with increased fNIRS-measured activation in the left IFG in the NH-VOC group. This expansion of brain activity to the IFG may be an attempt to compensate for the degraded speech signal or a neural correlate of the increased listening effort required to understand speech (e.g., Winn & Teece).

These findings highlight that distinct cortical activation patterns in specific brain regions are associated with speech recognition scores in adults CI listeners, with the STG, MTG, and IFG, particularly in the left hemisphere, playing key roles in this relationship. Some aspects of these findings remain inconclusive. This holds particularly true in the MTG and STG, where both a positive relationship (Lawrence et al. 2018; Levin et al. 2022) and a negative relationship (Steinmetzger et al. 2022; Zhou et al. 2018; Defenderfer et al. 2021) have been reported, but under different listening demands. Despite the limited number of studies and the inconclusive findings, a common observation is that fNIRS-measured cortical activities in the STG, MTG, and IFG are related to speech recognition scores in NH-VOC conditions, with the STG and MTG also having relationships among actual CI listeners. This indicates a potential application of monitoring the activities in these regions to predict speech recognition in adult CI listeners and whether they recruit other auditory-associated regions for processing and understanding speech.

### Individual differences in fNIRS-measured cortical activities and in speech recognition outcomes

While demonstrating a relationship between fNIRS speech-evoked activities and speech recognition scores in CI listeners is a valuable first step, it is also important to identify how much variance in speech recognition scores can be accounted for by speech-evoked activities. This understanding is important to enhancing fNIRS’s ability to detect individual differences in speech-evoked cortical activities among CI listeners and for improving its utility in assessing neural activity related to speech perception. For instance, if neural responses in certain brain regions can account for a significant portion of the variance in speech recognition among high-performing CI listeners, targeted interventions could be developed to enhance neural responses in these regions for lower-performing individuals. Therefore, we further analyzed studies with measures of speech perception and fNIRS responses to determine the extent to which individual variability in speech recognition scores among CI listeners can be attributed to differences in their speech-evoked cortical activities. Five studies reported a measure that allows us to examine this quality (Lawrence et al. 2018; Defenderfer et al. 2021; Steinmetzger et al. 2022; Levin et al. 2022; Zhou et al. 2018). In Steinmetzger et al. (2022), HbO amplitude in the STG of the implanted ears was negatively correlated with the percentage of correct words bilaterally, with a coefficient of determination (*R²*) of 0.23. This suggests that 23% of the variation in the percentage of correct words could be explained by variation in HbO amplitude in the STG. Zhou et al. (2018) found a negative correlation between HbO amplitude in the left STG with the percentage of words correct from a combination of consonant-nucleus-consonant (CNC) words and City University of New York (CUNY) sentences. The *R²* value was 0.42, suggesting that approximately 42% of the variation in the percentage of correct words could be explained by variation in HbO amplitude in the left STG. Levin et al. (2022) found that greater changes in HbO concentration in CI listeners, as indicated by higher mean beta values from General Linear Model (GLM) analysis of the HbO data, in response to listening to speech with speech-shaped noise at 0 dB signal-to-noise ratio (SNR), were associated with lower sentence recognition thresholds in noise. This relationship was observed in both the right and left MTG, with *R²* values of 0.25 and 0.13, respectively. Note that the negative relationship in this study means that greater MTG activation corresponded with better speech recognition (i.e., lower threshold). However, these results cannot be interpreted in terms of the extent to which variability in speech recognition scores in noise is explained by changes in HbO concentration because the *r* values in Levin et al. (2022) were calculated across all three groups of actual CI listeners, NH listeners, and NH-VOC condition, rather than focusing solely on actual CI listeners or a NH-VOC condition.

Two studies by Defenderfer et al. (2021) and Lawrence et al. (2018) in NH-VOC condition reported correlation coefficients describing the relationship between measures of speech perception and fNIRS activity. Defenderfer et al. (2021) found that HbO concentration in the left MTG was negatively correlated with the percentage of Hearing in Noise Test (HINT) sentences repeated back correctly (*R²* = 0.16), suggesting that approximately 16% of the individual differences in sentence recognition in noise can be attributed to variations in HbO concentration in the left MTG. In contrast, they observed a positive relationship in the left IFG (*R*^*2*^ = 0.19), suggesting that changes in HbO concentration in the left IFG can account for around 19% of variability in sentence recognition in noise in the NH-VOC condition. Lawrence et al. (2018) observed a positive correlation in the right STG between fNIRS response amplitude and the percentage of keywords correctly repeated in sentences (*R*^*2*^ = 0.27), such that approximately 27% of the variability in speech intelligibility can be explained by the fNIRS response amplitude in the right STG.

Overall, decreased fNIRS response amplitude in the left STG and the left MTG has been shown to account for approximately 16–42% of the variability in speech recognition in noise among actual CI listeners and in NH-VOC conditions (Steinmetzger et al. 2022; Defenderfer et al. 2021; Zhou et al. 2018). Conversely, elevated fNIRS response amplitude in the left IFG and right STG has been associated with increased speech recognition performance, explaining around 19% and 27% of the variability in speech recognition in noise in NH-VOC conditions (Defenderfer et al. 2021; Lawrence et al. 2018). These findings highlight how individual differences in speech perception among CI listeners can be differentially attributed to variations in fNIRS activity across different cortical regions, with greater speech recognition outcomes explained by reduced activation in some regions (i.e., the left STG and MTG; Steinmetzger et al. 2022; Defenderfer et al. 2021; Zhou et al. 2018) and by increased activation in other regions (i.e., the left IFG, right STG, and left and right Levin et al. 2022; Defenderfer et al. 2021; Lawrence et al. 2018). These mixed results highlight that, although certain patterns of cortical activity in specific brain regions are associated with speech recognition, the relationship may vary by region of interest (ROI), between actual CI listeners and the NH-VOC group, in its direction (positive or negative), and in the portion of variance explained by fNIRS measures. Overall, these findings support the use of fNIRS to explain speech recognition performance at the individual level at some point in the future contingent on further experimental and methodological improvements and considerations, which are discussed in the following sections.

## Major challenges

### Test-retest reliability

Understanding the challenges that fNIRS faces in studying speech-evoked neural activities in CI listeners can enhance its feasibility and utility in both research and clinical settings. Reliability is one of these challenges. While prior findings indicate that fNIRS is effective at identifying differences in group-level speech-evoked cortical activities, individual-level findings do not exhibit the same high level of test-retest reliability (Wiggins et al. 2016). Inconsistency becomes more important in CI listeners, as they already exhibit large individual differences in speech perception outcomes (Caswell-Midwinter et al. 2022; Holden et al. 2013). CI studies have captured some speech-evoked individual differences at the cortical levels (Defenderfer et al. 2021; Lawrence et al. 2018; Pollonini et al. 2014), which may be confounded, in part, by unreliable measurements. The measurement with low reliability presents a challenge for consistent interpretation by both researchers and clinicians. Therefore, it is essential to improve test-retest reliability among CI listeners so that fNIRS can be used to help enhance speech-perception outcomes. Employing standardized data processing methods and strategic montage design, such as those described in the following sections, can enhance the reliability of repeated measurements.

### Extracerebral noise

One issue that can hinder reliability is the presence of extracerebral noise in the fNIRS data. As with any non-invasive neuroimaging modality, using fNIRS requires retrieving data through the scalp, skull, and cerebrospinal fluid. This poses a challenge for fNIRS because the scalp has significant blood flow, and it can negatively influence the signal-to-noise ratio (SNR) of the data. The resulting physiological noise can completely obscure the findings of an experiment (e.g., van de Rijt et al., 2016), especially experiments on CI listeners which often involve measurement sensitivity to subtle changes in the speech signal, encoded by electrical pulses (Azadpour et al. 2018; Moberly et al. 2016). For this reason, it is important to develop and utilize a method that can reduce the impact of non-neuronal noise. One solution in the field is the use of short- channel regression, divided into a hardware and software steps (Brigadoi and Cooper 2015; Saager and Berger 2008; van de Rijt et al., 2016). Creating short channels in the montage involves placing some detectors nearer than usual to sources, typically maintaining an 8 to 15 mm separation in adults. These contrast with long channels, which are used to examine activation in the cortex and are typically spaced 30 mm to 40 mm apart on a standard atlas, although some may exceed a 45 mm separation (Zimeo Morais et al. 2018). During data processing, data obtained from the short channels can be used to determine and remove non-neuronal noise in a specified area of the scalp. Software and methods used for this process vary among prior studies on CI listeners (see Defenderfer et al. 2021; Levin et al. 2022; Pollonini et al. 2014; Shader et al. 2022; Steinmetzger et al. 2022; van de Rijt et al., 2016 for further details), but they all follow this general process. Using short channels has proven to be more effective than alternative noise removal methods that are based on the assumption that oxygenated and deoxygenated hemoglobin concentrations are anti-correlated (Zhou et al. 2021). While extracerebral noise poses an obstacle to fNIRS research, employing short-channel regression is recommended to effectively mitigate its impact.

### Natural listening conditions

Another challenge of using fNIRS in auditory neuroscience research is the difficulty or inability to replicate natural listening environments during experiments. This becomes particularly important when studying speech perception in CI listeners as their performance reduces significantly in background noise (Arjmandi et al. 2022a; Fetterman and Domico 2002; Fowler et al. 2021; Sladen and Zappler 2015), which frequently occurs in natural listening environments (Busch et al. 2017). Many paradigms utilize concatenated sentences or words, but these have been shown to only involve a subset of cognitive and neural mechanisms that are activated in real-world listening scenarios (Hickok and Poeppel 2000). Therefore, findings involving these types of stimuli may only be generalized to actual performance of CI listeners. Some methods have been proposed to address this challenge. One adopted by fNIRS studies in adult CI listeners is the use of speech-in-noise stimuli (Defenderfer et al. 2021; Levin et al. 2022). This approach is shown to better simulate the relatively noisy conditions people typically experience in natural listening environments. Another approach involves presenting stories in segmented series throughout the experiment to better simulate the attention dynamics observed in natural environments (e.g., Pollonini et al. 2014; Shader et al. 2022; van de Rijt et al., 2016). Although these methods replicate real-world listening scenarios, they diminish the feasibility of integrating controlled perceptual tasks and place greater emphasis on semantic understanding. Further advancements are necessary to maximize the overlap between laboratory findings and natural-listening cortical activation while controlling for those confounding factors.

### Montage design

Designing an effective montage is an important aspect of planning an fNIRS experiment, as it determines which ROIs are targeted and how efficiently their activation is captured. Early studies with adult CI listeners typically placed rectangular patterns of optodes over the auditory cortex, specifically targeting the STG (e.g., Olds et al. 2016; Pollonini et al. 2014) or utilize a small number of optodes to create a channel in a single ROI (e.g., van de Rijt et al., 2016). It was also common for these patterns to be condensed such that optodes were within close proximity of each other and could dually function to form long and short channels. Over time, there has been a shift towards employing less compact optode configurations in new montages (e.g., Defenderfer et al. 2021; Fullerton et al. 2023; Lawrence et al. 2018; Levin et al. 2022; Shader et al. 2022; Steinmetzger et al. 2022; Zhou et al. 2018), although Bortfeld (2019) discussed how some studies intentionally utilized higher-density montages to improve spatial resolution (Olds et al. 2016; Pollonini et al. 2014). It is of note that lower optode density did not inherently come with less channels, as new montages more frequently used dedicated short-channel detectors for regressing extra-cerebral noise (Defenderfer et al. 2021; Levin et al. 2022; Shader et al. 2022; Steinmetzger et al. 2022). These dedicated short-channel detectors are typically closer in proximity to the sources with which they form channels, in comparison to the short channels generated by non-dedicated detectors. This aligns with current evidence suggesting that 8.4 mm is typically an optimal separation for detecting hemodynamic fluctuations in the adult scalp (Brigadoi and Cooper 2015).

In addition, it has become more common for montages to use arrays with non-rectangular structure, indicating more specific targeting of desired ROIs (Levin et al. 2022; Shader et al. 2022; Steinmetzger et al. 2022; Zhou et al. 2018). One consistency across studies reviewed here is a 30 mm to 35 mm inter-optode separation for long channels. Optode count was also similar across experiments, as many of the more recent studies under review have utilized systems with approximately 32 optodes (Fullerton et al. 2023; Lawrence et al. 2018; Levin et al. 2022; Shader et al. 2022; Steinmetzger et al. 2022; Zhou et al. 2018), although studies prior to 2022 utilized systems ranging from 6 (van de Rijt et al., 2016) to 40 optodes (Olds et al. 2016; Pollonini et al. 2014). Researchers with access to a limited number of optodes may choose to examine only one hemisphere, as shown in Defenderfer et al. (2021). Consistency across studies is otherwise rare, as montages vary greatly based on research questions and target ROIs, and this variability necessitates a greater understanding of optimal methods for observing ROIs. A montage that fails to adequately capture data from target ROIs will potentially mislead audiences into assigning certain outcomes to incorrect regions. One challenge facing researchers is a lack of reporting specific locations of optodes in most studies. This complicates the replication of studies and hinders effective comparisons between them. Ideally, this important aspect of methodology would be reported in reference to a standardized atlas such as the ICBM-152 (Mazziotta et al. 1995, 2001a, b) or in another way that makes optode location clear and replicable. Enhanced transparency in fNIRS implementation among researchers will facilitate the sharing of knowledge, specifically in accurately collecting data from specified ROIs and investigating specific questions.

### Experimental design

The outcomes of speech-evoked fNIRS studies in CI listeners may be influenced by the design of experimental paradigms developed to address specific research questions. When examining the history of studies that use fNIRS to find cortical activity in response to listening or/and understanding speech, a few trends are present. First, some studies have included speech-in-noise in addition to or instead of speech-in-quiet recently (Defenderfer et al. 2021; Levin et al. 2022), possibly as part of the attempt to better simulate natural listening environments. It has also become more common to vary inter-stimulus intervals, a practice that is important for preventing the brain from time-locking hemodynamic responses based on expectation of stimuli (van de Rijt et al., 2016). Average interstimulus interval (ISI) duration has considerably varied across studies, ranging from 18 to 37.5 s in block designs (Fullerton et al. 2023; Levin et al. 2022; Olds et al. 2016; Shader et al. 2022; Steinmetzger et al. 2022; van de Rijt et al., 2016; Zhou et al. 2018) and 6.25 to 15 s in event-related designs (Defenderfer et al. 2021; Lawrence et al. 2018). Stimulus length for block-design studies varied between 7.5 and 20.5 s (Fullerton et al. 2023; Levin et al. 2022; Olds et al. 2016; Shader et al. 2022; Steinmetzger et al. 2022; van de Rijt et al., 2016; Zhou et al. 2018), and event-related designs employed stimulus lengths from under 2 to 5 s (Defenderfer et al. 2021; Lawrence et al. 2018). For block design, these stimulus lengths have tended to decrease over time. One important practice that is prevalent in some studies is the use of an attention task during the experiment (e.g., Fullerton et al. 2023; Levin et al. 2022; Shader et al. 2022). These tasks typically ask participants to answer a question related to the stimuli or give the participants context to help them understand a continuous story, and the tasks are frequent enough to keep the listener attentive but not so prevalent that they dramatically increase the duration of the study. These are particularly useful in paradigms that would otherwise just be passive listening because they give participants an incentive to focus. Each of these aspects of experimental design have been curated to better capture results over time, and adopting best practices will provide more reliable fNRIS study results in CI listeners.

## Potential clinical implications

Individual differences in speech recognition among CI listeners can be traced to issues with the peripheral processing of speech, specifically related to the electrode-neuron interface (e.g., Bierer, 2010), and differences in how the brain processes auditory information at the cortical level (Fallon et al. 2008; McKay 2018). Pinpointing the specific roles of peripheral factors, such as variations in the quality of ENI (Arjmandi et al. 2022a; DeVries et al. 2016; Goldwyn et al. 2010; MacDonald et al. 2011), as well as the cortical processing (e.g., Scheperle & Abbas, 2015) is essential for directing interventions effectively. For instance, a CI listener with high cortical processing ability of speech may be somewhat capable of compensating for the limited spectro-temporal resolution, primarily spectral resolution, in the transmission of speech cues and thus perform well in the identification of speech sounds. fNIRS is a valuable tool to distinguish the extent to which speech perception outcome is related to peripheral and cortical variations. Changes in cortical activities related to peripheral factors such as limited spectral resolution due to channel interaction may be localized to specific regions of the auditory cortex (Scheperle and Abbas 2015a, b). Cortical activities specific to central variations (e.g., neural adaptations, compensatory mechanisms) may involve a broader neural network or different brain regions or the connectivity between these regions. Identifying these brain regions and their activity is essential for pursuing how techniques such as auditory-verbal training (e.g., McKay 2018) and non-invasive neurostimulation (e.g., Mandalà et al. 2021) may be used to promote plasticity in speech-evoked cortical network and eventually improve speech recognition outcomes.

Patterns of speech-evoked cortical activities can be analyzed in combination with measures of peripheral processing such as measures of spectral resolution (e.g., psychophysical tuning curves and spectral ripple resolution; Anderson et al. 2011; DeVries and Arenberg 2018; Litvak et al. 2007; Won et al. 2007). This combined approach allows for the investigation of the extent to which variations in hemodynamic responses and speech recognition outcomes are linked to differences in peripheral processing. Additionally, it facilitates the examination of the degree to which these variations are attributable to compensatory processes occurring at the cortical level. Certain characteristics of speech-evoked cortical responses can be indicative of adaptive processes to understand how the cortex may adjust to challenges posed by degraded peripheral input (Alzaher et al. 2021; Fallon et al. 2008; McKay 2018). Understanding the contributions of peripheral and central factors to speech recognition outcomes in individual CI listeners can guide the development of personalized interventions. By understanding the relative contributions of these factors, researchers and clinicians can tailor strategies, including optimized CI signal processing, fine- tuned CI programming to address poor peripheral processing, and targeted auditory rehabilitation methods that engage cortical mechanisms to improve speech recognition outcomes.

## Discussions and future directions

Expanding the scientific and clinical community’s understanding of speech-evoked cortical activities measured by fNIRS may establish it as a unique choice for investigating CI listeners’ communication outcomes. Thus far, fNIRS has proven its ability to measure group-level cortical responses to speech stimuli in a variety of ROIs, especially the STG, MTG, and IFG (e.g., Defenderfer et al. 2021; Fullerton et al. 2023; Lawrence et al. 2018; Levin et al. 2022; Olds et al. 2016; Pollonini et al. 2014; van de Rijt et al., 2016; Steinmetzger et al. 2022; Zhou et al. 2018). Weaker speech-evoked neural activities reported in the MTG and STG in adult CI listeners compared to their NH peers indicate that this the restoration of speech processing through CI remains partial. The observed lower neural activities in these critical auditory processing regions suggests a potential degraded transmission of speech through CIs, which could be due to the limitations in processing spectral information (e.g., Fu et al. 1998; O’Neill et al. 2019; Winn and Litovsky 2015; Won et al. 2007). This implies that while cochlear implants offer significant access to speech sounds, the neural representation of speech lacks the richness and details available in normal hearing, emphasizing the importance of ongoing advancements in implant technology, CI programming strategies, and auditory rehabilitation approaches to enhance spectral fidelity and improve the overall quality of neural representation of speech sounds.

While these investigations in broad auditory regions have been important, few to no fNIRS studies have specifically targeted and analyzed some speech-related brain regions including the angular gyrus, supramarginal gyrus, planum temporale (Shader et al. 2022), fusiform gyrus (Levin et al. 2022), or Heschl’s gyrus (Shader et al. 2022) in adult CI listeners. Chen et al. (2022) did examine the angular gyrus, supramarginal gyrus, and fusiform gyrus in children, but it is beyond the focus of this review due to its study population. Targeting neural activity in these ROIs may be challenging due to their relatively deep location within the cortex, which makes it difficult because of the moderate spatial resolution of fNIRS (e.g., Stoppelman et al. 2013). This is particularly true for regions such as Heschl’s gyrus and the fusiform gyrus, which are moderately and deeply embedded in the cortex, respectively. Findings from current fNIRS studies have not yet shown consistent differences between CI and NH listeners in speech-evoked activation patterns, with mixed findings in the STG and relatively few studies in other ROIs.

The relationship between cortical activation patterns and speech recognition scores in adult CI listeners also plays a role in the knowledge framework needed to more effectively use fNIRS as a clinical and research tool. Prior findings from fNIRS studies indicate that decreased STG activity and increased LIFG and left fusiform gyrus activity are associated with increased speech recognition ability (Defenderfer et al. 2021; Levin et al. 2022; Steinmetzger et al. 2022), and findings in the MTG are mixed (Defenderfer et al. 2021; Levin et al. 2022). Results on the extent to which individual differences in fNIRS measures explain individual differences in speech recognition scores reveals varied findings across different brain regions. Individual CI differences in STG activity significantly accounted for variability in speech outcomes, explaining 23% (Steinmetzger et al. 2022) to 42% (Zhou et al. 2018) of the variance in correct word recognition. Decreased left MTG activity was found to account for 15.8% of the variance in sentence recognition in noise, while increased activity in the left IFG explained 18.5% of the variability in sentence recognition in noise (Defenderfer et al. 2021). Lawrence et al. (2018) found that 27% of the variability in speech intelligibility could be attributed to right STG activity. The differences in findings might be due to variations in study populations (actual CI versus NH-VOC condition), types of speech stimuli (ranging from spondee to vowels, words, and sentences), listening conditions (quiet versus noise), and the specific fNIRS measures used (e.g., raw HbO amplitude versus relative HbO amplitude between two conditions of speech intelligibility). Nevertheless, these findings indicate that variations in cortical activity of adult CI listeners measured by fNIRS are associated with the differences observed in speech recognition among CI listeners, with specific brain regions showing varied levels of involvement that either diminish or enhance speech perception abilities. Further studies are needed to clarify the direction and extent of these relationships across different brain regions, with particular focus on the STG and MTG as key brain regions involved in speech processing.

Regarding montage design, future studies are advised to employ short channels near 8.4 mm in separation, although temporal regions may be optimized at a different inter-optode separation due to their increased scalp thickness relative to other areas (Babiloni et al. 1997; Brigadoi and Cooper 2015). These experiments would also benefit from making stimuli better simulate CI listeners’ hearing experience in natural listening environments by employing speech in noise (Arjmandi et al. 2022a; Busch et al. 2017; Cucis et al. 2020; do Nascimento and Bevilacqua 2005; Hochberg et al. 1992; Whitmal et al. 2007) and experimenting with story- based stimuli when appropriate for a given research question. This is important as CI listeners have exhibited major difficulties understanding speech in a noisy and reverberant environment (e.g.Arjmandi et al. 2022a; do Nascimento and Bevilacqua 2005; Kressner et al. 2018). Another aspect of experimental design to consider is an attention task, which could be added to maintain focus in passive listening paradigms that otherwise lack interactivity. Each of these considerations will help researchers to more effectively leverage fNIRS for a nuanced understanding of how cortical activation correlates with speech perception in CI listeners.

Given that the major challenge in CI research is accurately and comprehensively understanding the source of large variability in speech outcomes, the enhancement of fNIRS-based studies is necessary for better capturing individual differences at the cortical level to make it practically applicable in clinical settings. Current research demonstrates that measures obtained from fNIRS can partially explain individual differences among CI listeners in speech recognition (Lawrence et al. 2018; Defenderfer et al. 2021; Steinmetzger et al. 2022; Levin et al. 2022; Zhou et al. 2018). However, there are only three studies specifically involving actual CI listeners (Steinmetzger et al. 2022; Levin et al. 2022; Zhou et al. 2018) while Levin et al. (2022) included the data from all NH, CI, and NH-VOC groups in their analysis. This may obscure specific patterns or associations that could be unique to CI listeners, as their neural processing and speech perception mechanisms are not necessarily the same as those of NH individuals and even NH-VOC condition. Future studies on individual differences in speech-evoked activity in actual CI listeners are warranted to verify these findings.

Although there are many strides that still need to be made in using fNIRS for auditory neuroscience research and with CI listeners, substantial progress has been made thus far. The techniques explained in this review have collectively increased the feasibility of measuring individual differences. Despite this, some challenges remain as discussed in this review, and future studies should prioritize addressing them to enhance the effectiveness of fNIRS in both research and clinical settings. The field may also benefit from combining fNIRS and EEG to enhance capturing speech-evoked neural activities in CI listeners by integrating the moderate spatial resolution of fNIRS with the high temporal resolution of EEG. In addition, further analysis of regions that have been extensively studied, as well as new research in regions outside the STG, MTG, and IFG, would provide specific, valuable information about speech-evoked neural networks in CI listeners. Alongside these advances, ensuring the accessibility of information related to fNIRS setup and analysis is important for facilitating collaboration and finding consensus on fNIRS implementation. Continuous progress in these areas could establish fNIRS as a reliable clinical tool for assessing cortical contributions to perceptual outcomes in individual CI listeners, leading to more effective and personalized solutions for their speech perception challenges. It would also allow for the evaluation of CI signal processing, programming strategies, and auditory rehabilitation approaches in enhancing cortical processing of speech.

Table 1. Summary of fNIRS studies on speech-evoked cortical activities in adults CI listeners. Information includes experiment design, type of speech stimuli, listening conditions (quiet or noise), participants information, fNIRS setup, regions of interest, behavioral measure of speech perception, and key findings. CI: cochlear implant; fNIRS: functional near-infrared spectroscopy; NH: normal hearing; NH-VOC: normal hearing’s response to vocoded speech; HINT: Hearing in Noise Test; BKB: Bench-Kowal-Bamford; CNC: Consonant-Nucleus-Consonant; CUNY: City University of New York; OLSA: Oldenburg Sentences Test; SRT: Speech Recognition Threshold; STG: Superior Temporal Gyrus; DLPFC: Dorsolateral Prefrontal Cortex; MTG: Middle Temporal Gyrus; IFG: Inferior Frontal Gyrus; MFG: Middle Frontal Gyrus; STS: Superior Temporal Sulcus; PrG: Precentral Gyrus; PoG: Postcentral Gyrus; IPL: Inferior Parietal Lobe; HbO: oxygenated hemoglobin response, HbR: deoxygenated hemoglobin response.

## Data Availability

The data and materials will be available upon request from the corresponding author.
